# Impact of age on unicompartmental knee arthroplasty outcomes: a systematic review and meta-analysis

**DOI:** 10.1007/s00167-022-07132-x

**Published:** 2022-09-05

**Authors:** Loay A. Salman, Abedallah Abudalou, Harman Khatkar, Ghalib Ahmed, Stephanie G. Dakin, Benjamin Kendrick, David W. Murray

**Affiliations:** 1 Present Address: Nuffield Department of Orthopaedics, Rheumatology and Musculoskeletal Sciences, Botnar Research Centre, University of Oxford, Windmill Road, Oxford, OX3 7LD UK; 2 Orthopaedics Department, Hamad General Hospital, Hamad Medical Corporation, PO Box 3050, Doha, Qatar

**Keywords:** Unicompartmental knee arthroplasty, Age, Revision, PROMs

## Abstract

**Purpose:**

Unicompartmental knee arthroplasty (UKA) is an effective treatment for late knee osteoarthritis (OA). Young age (< 60 years) has been associated with worse outcomes. The goal of this systematic review and meta-analysis is to study the effect of age on UKA outcomes.

**Methods:**

The primary objective was to compare the UKA revision rate in young patients with that of old patients, using the age thresholds of 60 and 55 years. Secondary objectives were patient-reported outcome measures (PROMs) and implant design. Five databases were searched in December 2021 for original comparative studies with a minimum of 1-year follow-up. No restrictions were placed on the type of UKA prosthesis.

**Results:**

A total of 11 observational studies with 6130 knees were included. A mean MINORS score of 19 was assigned to the review. The mean age of patients was 64 years, with average follow-up of 7.5 ± 2.98 years. There was no significant difference in revision rate, incident or PROMs between young and old patients in the analysis for each age threshold. Further sub-analysis adjusting for implant type in mobile- and fixed-bearing prostheses also showed similar results between those above and under 60 and 55 years.

**Conclusion:**

Young age was not associated with a higher revision rate or lower functional scores. Thus, this review provides evidence that age alone is not a contraindication to UKA, but the surgical choice must be based on several elements, and this finding should be applied in context, given the binary division and heterogeneity of patients.

**Level of evidence:**

III.

## Introduction

Although indications for unicompartmental knee arthroplasty (UKA) remain controversial, UKA contributed to 11% of all primary knee replacements in the UK in 2019 [5, 35]. UKA is a minimally invasive procedure that preserves normal knee kinematics and if performed well, offers excellent 15-year survivorship of over 91% [32]. Furthermore, TOPKAT, a large multicentric randomized controlled trial, has also found a similar revision rate in both UKA and TKA (0.75, 0.37–1.53; *p* = NS) and comparable OKS (mean difference 1.04, 95% CI –0.42 to 2.50; *p* = NS) at 5-year follow-up [3], advocating the use of UKA as a first choice when properly indicated.

In their landmark paper published in 1989, Kozinn and Scott [18] proposed strict criteria for UKA that deemed patients younger than 60 not ideal candidates for the procedure. In contrast, subsequent studies [25, 33] advocated performing UKA based on pathoanatomy, particularly in mobile-bearing UKA, and disregarded the age criterion as an unnecessary contraindication.

Conflicting findings from registry data and other studies exist regarding the effect of age on UKA outcomes [15, 32, 35]. There are currently no systematic reviews assessing this relationship within the UKA cohorts; therefore, such a review, which is the first to investigate all non-registry data, would help guide surgical management and inform how surgeons select those patients that are likely to benefit from UKA.

This review aims to investigate how patient’s age affects revision rate and patient-reported outcome measures (PROMs) following UKA by comparing patients older than 60 with patients younger than 60. In addition, a subgroup analysis for the cut-off age of 55 to further assess the optimal age threshold for UKA candidacy was performed. Additional analysis of how age affects fixed- and mobile-bearing prostheses was done. Thus, the null hypothesis is that there is no significant difference in revision rate and functional outcome scores between younger (< 60 years) and older (> 60 years) patients undergoing UKA.

## Methods

This systematic review was conducted in line with the Preferred Reporting Items for Systematic Reviews and Meta-Analyses (PRISMA) guidelines [24]. To ensure transparency and efficiency, a protocol registration was sought in advance on the International Prospective Register of Systematic Reviews (PROSPERO) with the registration number: CRD42021248322.

### Search strategy

PubMed/Medline, Ovid, Web of Science, Google Scholar and Cochrane library databases were searched until December 2021 with the following keywords and their derivatives: “age”, “unicompartmental”, “unicondylar”, “knee”, “arthroplasty”, “replacement”, “UKA”, “UKR”, “revision”, and “outcome”—see appendices for detailed examples of database searches. Two authors independently screened the search results based on the title and/or abstract. Conflicts were resolved via a discrepancy meeting with a third, more senior author. A full-text review of articles that met the eligibility criteria was performed, and references of included articles were manually sought to ensure all relevant studies were included.

### Outcomes of interest

In this review, revision is the primary outcome and is defined as “Any operation performed to add, remove or modify one or more components of a joint replacement” [35]. Functional outcome measures, including the Knee Society Score (KSS) [28] and Oxford Knee Score (OKS) [26], were used as secondary outcomes of interest to compare younger and older patients undergoing UKA.

### Eligibility criteria

Studies comparing the revision rate and PROMs (KSS, OKS) between younger patients (< 60 years) and older patients (> 60 years) following UKA were included. Also, studies in which these two UKA outcomes were compared in patients younger than 55 and in patients older than 55 were included to explore further the influence of different age groups on UKA outcomes. No restrictions were placed on the type of UKA prosthesis. Exclusion criteria entailed noncomparative studies, reviews, and studies with short follow-up of less than one year, or unextractable data needed for analysis. Table 1 summarizes the complete list of inclusion and exclusion criteria.

**Table 1:** Summary of eligibility criteria

Inclusion criteria
1. Studies comparing revision of UKA between young (< 55, < 60) and old (> 55, > 60) age groups
2. Studies comparing functional scores (KSS, OKS) of UKA between young (< 55, < 60) and old (> 55, > 60) age groups 3. Studies with minimum follow-up period of 1 year
4. Comparative RCT and observational studies stratifying the study population by age
5. Studies reporting medial UKA or mainly medial UKA procedures
6. All types of UKA prosthesis designs
Exclusion criteria
1. Studies with different indications for UKA than OA
2. Noncomparative or not reporting outcomes or failures by subgroups (i.e., young vs old)
3. Review articles, cross-sectional, case series and reports
4. Preclinical studies
5. Studies with registry, incomplete or unextractable data
6. Studies published in languages other than English

**Fig. 1:**
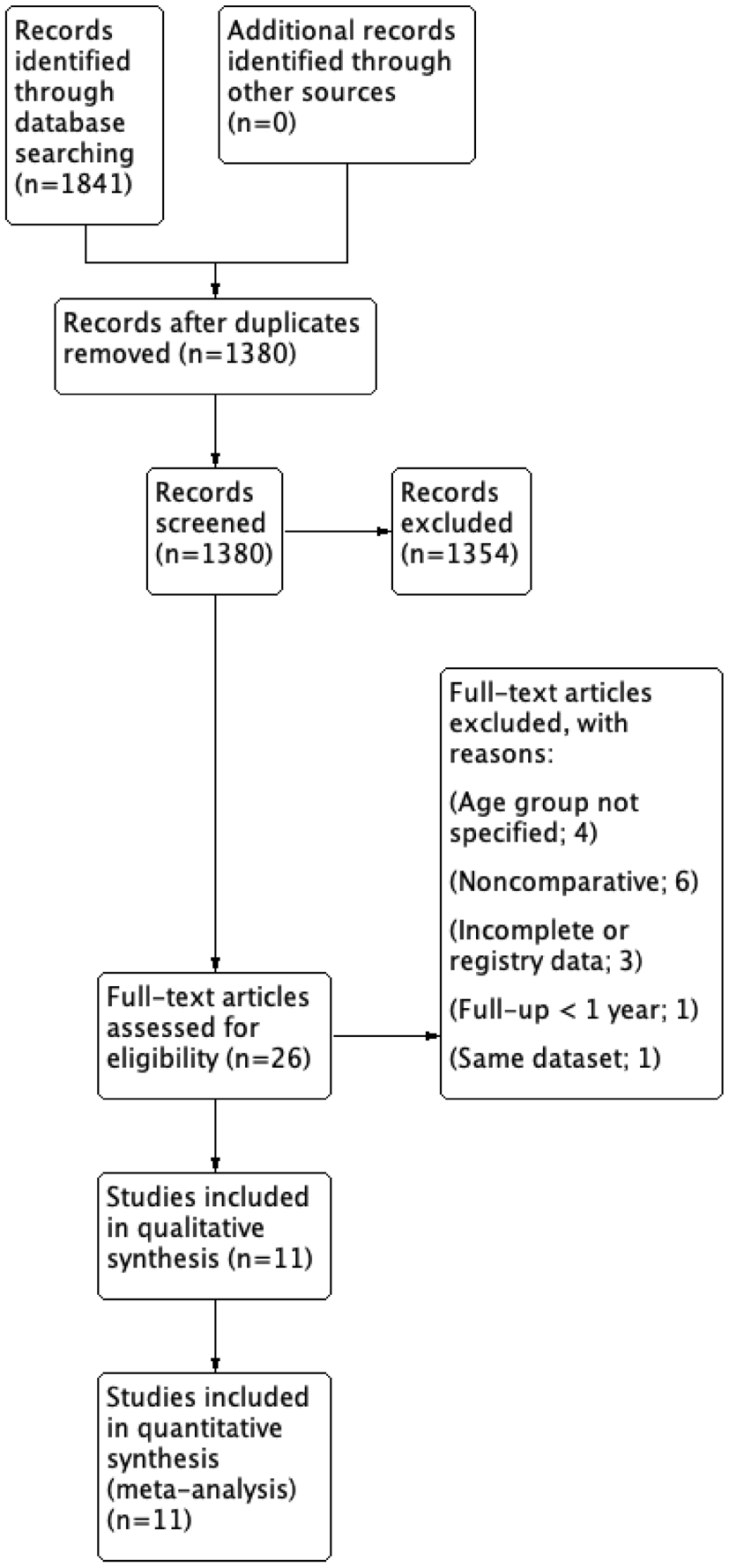
PRISMA flow diagram of record identification, screening and selection in meta-analysis

Rayyan AI website was used to manage search results [31]. Searching the databases identified 1841 papers, and after removing 361 duplicates, 1380 records were screened by title and abstracts, of which 1354 were excluded. A total of 26 papers were eligible for a full-text review. As a result, 11 studies met the eligibility criteria and were included in the qualitative and quantitative synthesis (Fig. 1).

### Data extraction and items

Two independent reviewers used a pre-designed data collection sheet (Appendices) in Microsoft Excel to extract data. The extracted demographic data included the first authors’ surnames, study year, design and location, prosthesis type, mean age of patients, number of participants and knees, age groups, follow-up time, number of revisions, functional outcomes, statistical tests and conclusion.

### Qualitative assessment

The risk of bias for each study was assessed using the Methodological Index for Non-Randomized Studies (MINORS) criteria [37]. Furthermore, for each study an Oxford Centre for Evidence-Based Medicine (OCEBM) [30] level of evidence (LoE) was assigned and then attributed an overall GRADE recommendation to the whole review, per the Cochrane collaboration handbook.

### Quantitative analysis

The quantitative synthesis of data was performed using REVMAN 5.4 (Cochrane Collaboration, Copenhagen) review manager [36] and STATA statistical software [38]. The revision outcome was assessed using two methods; first, the raw number of revisions (incident) was calculated to compare between both age groups. Additionally, for the annual revision rate, revisions per 100 observed component years (CY) was adopted [11]; a widely accepted method in orthopedic literature and is used to overcome inconsistencies in data sources and follow-up periods across studies and ensure appropriate weighting in comparisons.

Odds ratio (OR) and 95% confidence interval (CI) for dichotomous variables (revision incident) and the means difference for continuous variables (annual revision rate and PROMS) were calculated. Mean and standard deviations were calculated using Poisson distribution. A *p* value less than 0.05 was considered significant. A random-effect model was utilized, and heterogeneity was quantified with the *I*^2^ statistic test. A sub-analysis based on prosthesis type (fixed or mobile-bearing) to explore the reasons for heterogeneity was performed.

## Results

### Study characteristics

Among the 11 included studies, 6 were used to compare how age affects UKA outcomes in patients younger than 60 and patients older than 60 years. The other five studies compared patients younger than 55 with patients older than 55 years. Moreover, three studies generated a meta-analysis of KSS functional outcomes. Five studies (45%) were retrospective, and six (55%) were prospective cohorts. There were no randomized controlled trials retrieved. A total of 6,130 knees were included, with a mean patient age of 64 ± 10.1 (Table 2).

**Table 2:** A summary of baseline study characteristics, FU (Y): follow-up in years

Author, year	Design, LoE	Country	UKA design	No. ofpatients	No. of knees	Age categories	FU (Y)	Outcomes	Conclusion
Price [34]	Retrospective, 3a	UK	Mobile	447	564	< 60, > 60	10	Revision, HSS	Revision: comparable PROM: ↑ in young
Collier [4]	Retrospective, 3a	USA/Japan	Fixed	NR	245	45–59, 60–69, 70–79, 80–89	9	Revision	Revision: ↑ in young
Kort [16, 17]	Cohort, 2b	Netherlands	Mobile	185	200	< 60, > 60	4.5	Revision KSS, WOMAC, SF- 36	Revision & PROM: comparable
Ingale [13]	Retrospective, 3a	UK	Mobile	465	527	< 60, 60–69, 70–79, > 80	4.23	Revision KSS	Revision: ↓ In old PROM: comparable
Kristensen [19]	Cohort, 2b	Denmark	Mobile	579	695	< 60, > 60	4.6	Revision	Comparable
Hamilton [10]	Cohort, 2b	UK	Mobile	818	1000	< 60, > 60	10	Revision KSS, OKS, Tenger	Revision and PROM: comparable
Kennedy [15]	Cohort, 2b	UK	Mobile	818	1000	< 55, 55–65, 65–75, > 75	10	Revision OKS	Comparable
Lee [20]	Retrospective, 3a	Singapore	Fixed	142	142	< 55, > 55	13	Revision OKS, KSS	Revision and PROM: comparable
Venkatesh [39]	Cohort, 2b	UK	Fixed	148	175	< 55, > 55	5.6	Revision KSS	Revision and PROM: comparable
Wang [40]	Retrospective, 3a	China	Mobile	500	582	< 50, 55–70, > 70	5.27	Revision OKS, KSS	Revision and PROM: comparable
Mohammad [23]	Cohort, 2b	UK	Mobile	870	1000	< 55, 55–65, 65–75, > 75	6.5	Revision OKS, KSS	Revision and PROM: comparable

### Risk of bias and level of evidence (LoE)

Risk of bias assessment produced an average MINORS score of 19, ranging from 17 to 22 (Table 3). Based on the OCEBM criteria, six studies were level 2b, and five were level 3b (Table 2), with an overall grade B of recommendation assigned to the review. This grade indicates the consistency of findings across included studies and denotes that this piece of evidence can be recommended and generally followed in clinical practice [8].

**Table 3:** MINORS criteria

Study	A clearly stated aim	Inclusion of consecutive patients	Prospective collection of data	End points appropriate to the aim of the study	Unbiased assessment of the study end points	Follow-up period appropriate to the aim of the study	Loss to follow-up less than 5%	Prospective calculation of the study size	An adequate control group	Contemporary groups	Baseline equivalence of groups	Adequate statistical analysis	Total
Price 2005	2	0	1	1	0	2	2	2	1	2	2	2	17
collier 2006	2	0	0	2	1	1	2	1	2	2	2	2	17
Kort 2007	2	1	2	2	0	2	2	1	2	1	1	1	17
Ingale 2013	2	0	2	2	1	2	2	2	2	2	1	2	20
Kristensen 2013	2	2	2	2	0	2	1	2	2	2	1	2	20
Hamilton 2017	2	2	2	2	1	2	0	2	2	2	2	2	21
Kennedy 2018	2	2	2	2	2	2	0	2	2	2	2	2	22
Venkatesh 2019	2	2	2	2	0	2	1	2	1	2	1	2	19
Lee 2019	2	0	2	2	1	2	1	2	2	2	1	2	19
Wang 2021	2	0	1	2	2	2	1	0	2	2	1	2	17
Mohammad 2021	2	1	2	2	1	2	2	2	2	2	2	2	22

#### Revision incident

**Fig. 2:**
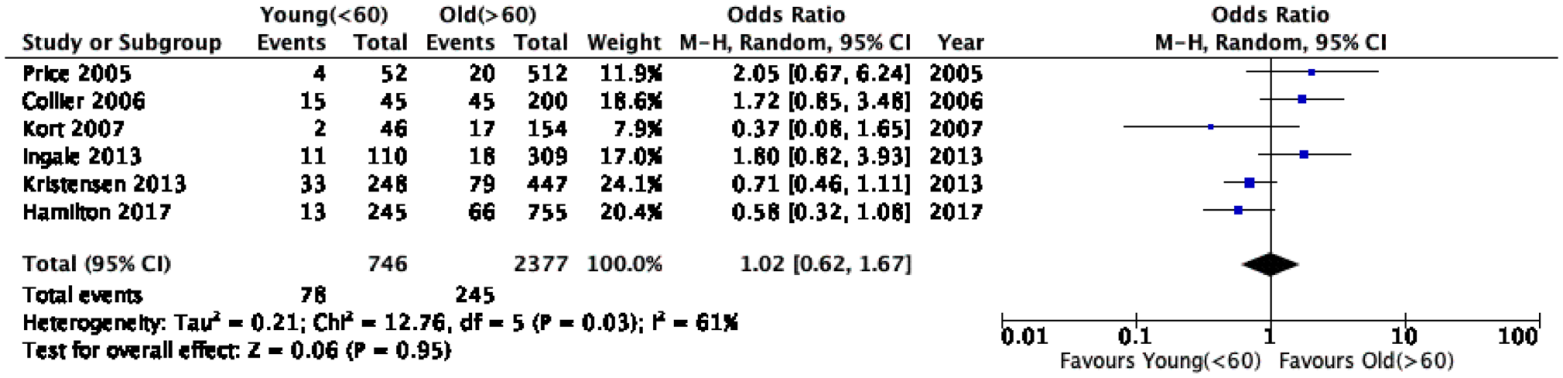
Comparison of the revision incidents (events) between young (< 60 years) and old (≥ 60 years) patients. *CI* confidence interval

**Fig. 3:**
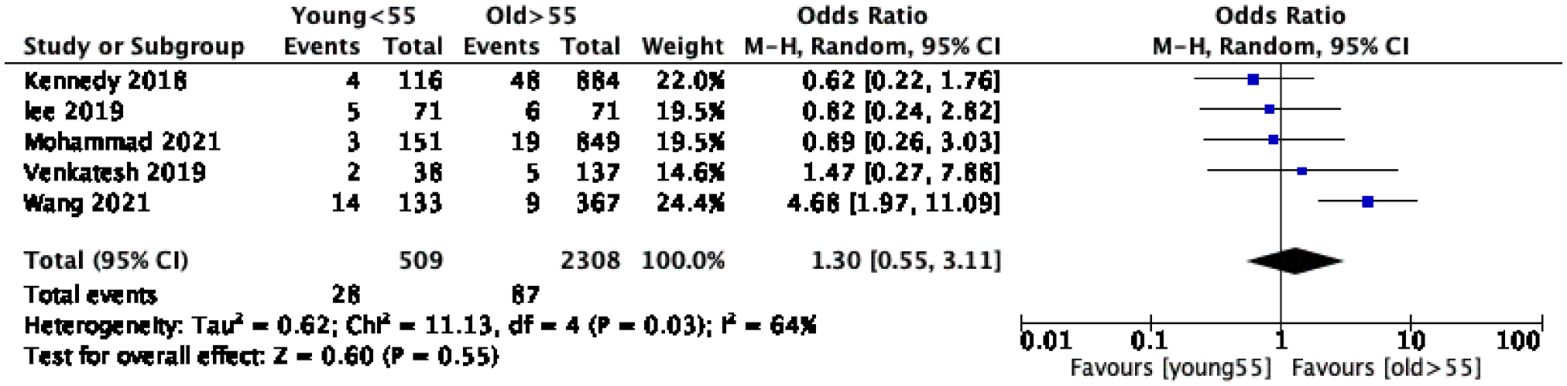
Forest plot comparison of the revision incidents between young (< 55 years) and old (≥ 55 years) patients. *CI* confidence interval

The overall number of UKA revisions was 438, of which 106 were at the young age groups and 332 were in the older age groups. Analysis of studies of 60-year-olds revealed a nearly equivalent risk of revision in both age groups with an OR of 1.02 (95% CI 0.62, 1.67) and moderate heterogeneity (*I*^2^ = 61%) (Table 4). However, this was not statistically significant (*p* = 0.95) (Fig. 2). On the other hand, UKA revisions were 1.3 times (95% CI 0.55, 3.11) more likely in patients younger than 55 compared to those older than 55 years. This effect was not statistically significant (*p* = 0.55, *I*^2^ = 64%) (Fig. 3).

**Table 4:** Meta-analysis summary of primary outcomes for revision and secondary outcomes using both age cutoff of 60 and 55 years

Outcome	Number of knees	Number of events (CY)	Heterogeneity (*I*^2^)	*P* value	OR or MD (95% CI)
Overall revision rate (age < 60, age > 60 years)	3123	(5188, 18,526)	97%	NS	MD − 0.05 (− 0.68, 0.57)
Overall revision rate (age < 55, age > 55 years)	2817	(3765, 17,770)	97.57%	NS	MD 0.29 (− 0.39, 0.96)
Revision incidents (Age < 60, age > 60 years)	3123	323	61%	NS	OR 1.02 (0.62, 1.67)
Revision incidents (Age < 55, age > 55 years)	2817	115	64%	NS	OR 1.30 (0.55, 3.11)
Revision rate (mobile bearing, age < 60, age > 60 years)	2878	263	97%	NS	MD − 0.28 (− 0.93, 0.38)
Revision rate (Fixed bearing, age < 55, age > 55 years)	317	18	73%	NS	MD 0.06 (− 0.37, 0.49)
KSS (age < 60, age > 60 years)	1600	–	96.59%	NS	MD 6.69 (− 7.05, 20.43)
OKS (age < 55, age > 55 years)	2142	–	–	–	Mean scores 32, 34

#### Revision rate

**Fig. 4:**
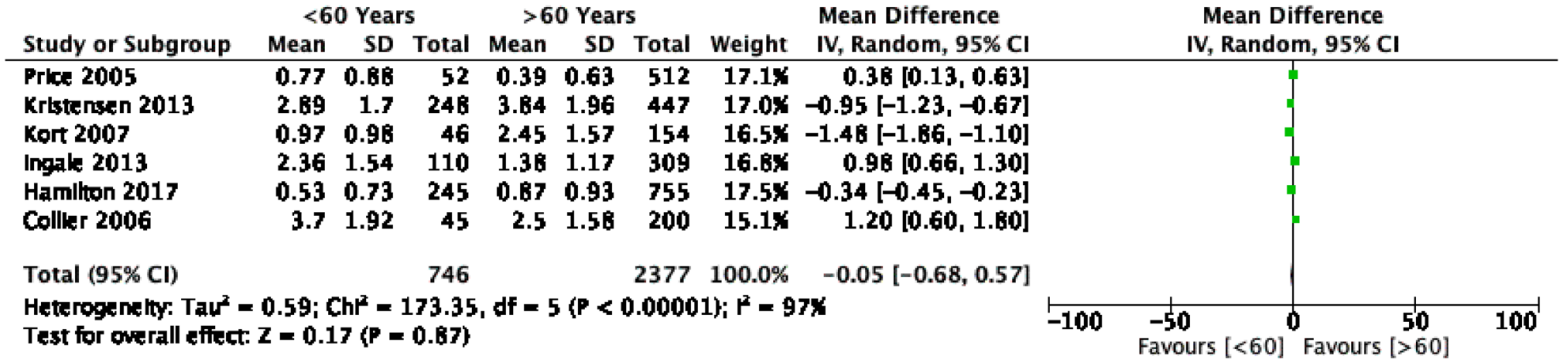
Forest plot comparison of the overall revision rate between young (< 60 years) and old (≥ 60 years) patients. *CI* confidence interval

**Fig. 5:**
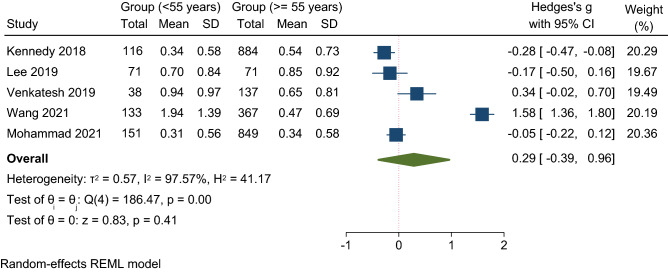
Forest plot comparison of the overall revision rate between young (< 55 years) and old (≥ 55 years) patients. *CI* confidence interval

In studies of 60-year-olds (Fig. 4), the calculated annual revision rate was insignificant in patients younger than 60 years, with a mean difference of 0.05% per annum compared with that of patients older than 60 years (*p* = 0.87). In contrast, analysis of the studies of 55-year-olds (Fig. 5) yielded an annual revision rate 0.29% higher in patients younger than 55 years than in patients older than 55 years. Again, this result was not statistically significant (*p* = 0.41) with 95% CI − 0.39, 0.96.

Furthermore, subgroup analyses adjusting for the type of UKA design demonstrated no significant differences between age groups in 2878 mobile-bearing (NS) and 317 fixed-bearing (NS) prostheses and gave revision rates of 0.28% pa and 0.06% pa higher in older patients for each design, respectively (*I*^2^ > 92%). Table 5 highlights the exact component years and annual revision rate across age groups in each study.

**Table 5:** Observed component years and total annual revision rate comparing younger vs older age groups

Study	Year	Observed component years		Annual revision rate %	
		Age < 60 years	Age > 60 years	Age < 60 years	Age > 60 years
Price	2005	520	5120	0.77	0.39
Collier	2006	405	1800	3.70	2.50
Kort	2007	207	693	0.97	2.45
Ingale	2013	465.3	1307.07	2.36	1.38
Kristensen	2013	1140.8	2056.2	2.89	3.84
Hamilton	2017	2450	7550	0.53	0.87

		Age < 55 years	Age > 55 years	Age < 55 years	Age > 55 years
Kennedy	2018	1160	8840	0.34	0.54
Lee	2019	710	710	0.70	0.85
Venkatesh	2019	212.8	767.2	0.94	0.65
Wang	2021	700.91	1934.09	1.94	0.47
Mohammad	2021	981.5	5518.5	0.31	0.34

#### KSS and OKS

**Fig. 6:**
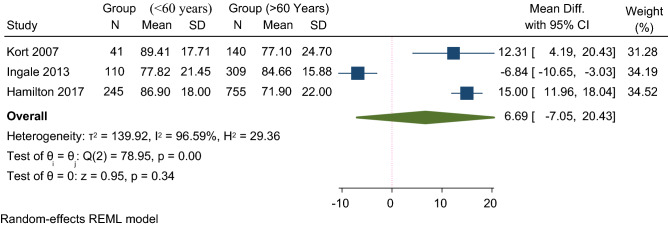
Forest plot comparison of mean KSS between young (< 60 years) and old (≥ 60 years) patients. *CI* confidence interval

Seven studies have reported a KSS outcome (Table 2), all of which demonstrated no statistical difference between younger and older age groups, with a trend towards better function in the young. The quantitative analysis of KSS in three studies showed a higher KSS score in the older than 60 age group, with a 6.69 mean difference in 95% CI (Fig. 6). However, this difference was insignificant and likely due to chance (*p* = 0.34). Five studies reported OKS(Table2), three of which were eligible for descriptive analysis [15, 20, 23] and showed comparable results with a mean score of 32.07 ± 9.9 in patients younger than 55 years and 34.03 ± 10 in patients older than 55 years at 10-year follow-up.

## Discussion

In agreement with the null hypothesis, the main finding of this systematic review and meta-analysis is that the risk of UKA revision is not associated with age, as there was no significant difference observed in UKA outcomes between young patients and old patients. To demonstrate this, a comparison between patients younger than 60 and patients older than 60 was done, and in the older age group, the revision incident was equivalent, and the annual revision rate was 0.05% higher (0.5% at 10-year follow-up). Further analysis of the 55-year age threshold yielded results favouring patients older than 55: in patients younger than 55, the revision incident was 1.3 times higher, and the mean revision rate was 0.29% higher (2.9% at 10-year follow-up). However, these results were statistically insignificant (NS), which reflect comparable outcomes in both age groups.

Contrary to our and others findings, data from national registries [1] report a higher risk of revision (2.9 times) in the younger population, particularly in those aged less than 55 years. Kennedy et al. [15] attributed this observation to the variability in UKA indications adopted in registry studies *versus* non-registry studies. While UKA indications were mainly based on pathoanatomy [6] in database studies, registry data have little information on indications and techniques, which makes UKA candidacy assessment even more challenging [15, 33]. There is evidence that the premature performance of UKA with only partial cartilage thickness loss might be linked to the higher revision rate and poor outcomes in younger patients [9, 27]; however, if appropriately indicated in anteromedial bone-on-bone arthritis, UKA should have an equivalent if not lower revision rate than primary TKA [14, 15].

The qualitative analysis of seven studies reporting KSS and five studies reporting OKS showed no difference across various age groups. A limited analysis of three studies showed an insignificant difference (*p* = 0.34) of KSS score favouring patients older than 60 with a mean difference of 6.69. UKA is a minimally invasive surgical procedure characterized by the preservation of knee ligaments [29]. A substantial improvement in postoperative functional outcomes is usually seen, irrespective of age. As a result, higher PROMs are expected and likely attributed to the restoration of normal knee kinematics and leg alignment, particularly in younger patients, due to arthritic pain reduction [7, 15].

UKA outcomes can be influenced by type, design and implant properties [2]. Our review includes studies of mixed UKA designs, and we observed no statistical difference in the mobile- and fixed-bearing prosthesis revision rate between the young group and old group. This finding is supported by a recent meta-analysis of 17 studies (2612 knees) that compared mobile- and fixed-bearing UKAs and showed no difference in revision (OR = 0.96; 95% CI 0.63–1.46; *p* = 0.85) and radiological outcomes [41]. Another meta-analysis of 1,996 knees showed comparable survivorship and revision incident of 1.22 (95% CI: 0.83–1.80, *p* = 0.32) [12]. In contrast, a higher risk of polyethylene wear and aseptic loosening is evident in the fixed-bearing design [15]. In addition, a recent level III systematic review evaluated the effect of cementing in 10,736 UKAs and found that cementless UKA was associated with lower revision rate than the cemented UKA (0.45% pa vs 0.73% *per annum*, p < 0.001). However, this difference was insignificant (*p* = 0.41) in the non-Oxford UKAs with similar revision rates of cemented and cementless UKRs (0.57% pa vs 0.69% pa) [22].

As the effect of patient age at the time of operation on UKA outcomes remains highly debatable with conflicting findings and no consensus in the literature, this systematic review provides the most inclusive and highest evidence on the topic. As a result, a lower threshold for performing UKA in the younger population might be expected at the expense of TKA when indicated, regardless of the age factor. This shall delay the need for TKA in the appropriate candidates and reduce the associated health costs.

Although this review has many strengths, several limitations must be acknowledged. First, the review outcomes had moderate to substantial heterogeneity. This heterogeneity is probably related to the binary analysis of two dichotomous age categories (i.e. below and above 60 and 55 years). A better approach would be to compare more precise age groups: for example, younger than 40, 40–45, 45–50, 50–55, 55–60 years, and so on. However, due to limited studies and the inconsistent reporting of age in the literature, the exploration of only the 55-year and 60-year age cutoff points were possible. Hence, this heterogeneity was accepted based on the nature of the review and age being an inconsistent and non-binary variable. Therefore, cautious interpretation of the results of this review in clinical practice is warranted.

Considering that UKA surgery has a learning curve [21], another weakness was the inadequate reporting of such factors as surgeon/hospital volume and surgical technique. Also, matching for patient’s weight, activity level, and baseline comorbidities was not clearly stated in all studies. Cohort and retrospective studies were included, as they represented the highest available level of evidence. Future prospective studies, ideally multicentric, are needed to adjust for these confounders.

Finally, 8 out of 11 included studies used mobile-bearing UKA, with bigger sample sizes and longer follow-up, suggesting that our finding could be more representative in this implant type. However, the presence of at least three studies using fixed-bearing design enhanced the external validity and generalizability of our results.

## Conclusion

This meta-analysis aimed to assess the impact of age on UKA outcomes by comparing younger age groups with older ones using 60-year and 55-year age thresholds. An equivalent revision rate between patients older than or younger than 60 and 55 years was demonstrated. Also, a comparable result between both arms of the study was observed with PROMs, namely KSS and OKS. Nevertheless, this evidence supports the finding that young age alone (below 60 years) should not be considered suboptimal in UKA.

## Searching strategy examples: Database

**(**1) Medline (Ovid MEDLINE® Epub Ahead of Print, In-Process & Other Non-Indexed Citations, Ovid MEDLINE® Daily and Ovid MEDLINE®) 1946 to present. *n* = 831.

**Table:**

#	Query	Results from 1 Dec 2021
1	age.mp. [mp = title, abstract, original title, name of substance word, subject heading word, floating sub-heading word, keyword heading word, organism supplementary concept word, protocol supplementary concept word, rare disease supplementary concept word, unique identifier, synonyms]	9,156,352
2	young*.mp. [mp = title, abstract, original title, name of substance word, subject heading word, floating sub-heading word, keyword heading word, organism supplementary concept word, protocol supplementary concept word, rare disease supplementary concept word, unique identifier, synonyms]	1,528,140
3	old*.mp. [mp = title, abstract, original title, name of substance word, subject heading word, floating sub-heading word, keyword heading word, organism supplementary concept word, protocol supplementary concept word, rare disease supplementary concept word, unique identifier, synonyms]	1,547,553
4	1 or 2 or 3	9,920,818
5	*Arthroplasty, Replacement, Knee/	22,839
6	(unicompartmental knee arthroplasty or unicondylar knee arthroplasty or partial knee replacement).mp. [mp = title, abstract, original title, name of substance word, subject heading word, floating sub-heading word, keyword heading word, organism supplementary concept word, protocol supplementary concept word, rare disease supplementary concept word, unique identifier, synonyms]	1,772
7	("UKA" or "UKR").mp. [mp = title, abstract, original title, name of substance word, subject heading word, floating sub-heading word, keyword heading word, organism supplementary concept word, protocol supplementary concept word, rare disease supplementary concept word, unique identifier, synonyms]	1,510
8	6 or 7	2,112
9	5 and 8	1,448
10	outcome*.mp. [mp = title, abstract, original title, name of substance word, subject heading word, floating sub-heading word, keyword heading word, organism supplementary concept word, protocol supplementary concept word, rare disease supplementary concept word, unique identifier, synonyms]	2,656,185
11	"revision*".mp. [mp = title, abstract, original title, name of substance word, subject heading word, floating sub-heading word, keyword heading word, organism supplementary concept word, protocol supplementary concept word, rare disease supplementary concept word, unique identifier, synonyms]	103,997
12	10 or 11	2,725,609
13	4 and 9 and 12	831

(2) Web of science: *n* = 762

(3) Cochrane Library: *n* = 134 “unicompartmental knee arthroplasty” OR “unicondylar knee arthroplasty” OR “partial knee replac*” OR “UKA” OR”UKR” AND “age” OR “young*” OR “old*” AND “revision”

## “PICOs” framework adopted

**Table:**

Population	Inclusion: adult patients > 18 years old, undergoing primary UKA indicated primarily for medial OA. Regardless of gender, BMI and ethnicity Exclusion: patients under 18 years, undergoing TKA, secondary UKA or having UKA for lateral compartment OA
Intervention	Primary UKA in younger population Below 60 years Below 55 years (sub-analysis)
Comparators	Primary UKA in older population Above 60 Y Above 55 Y (sub-analysis)
Outcomes	Primary outcome: revision rate (CY) Secondary outcomes Revision incidents Patient-reported outcome measures: KSS, OKS

## Data collection form

**Table:**

Study
Year of publication
Country of study origin
Study design, OCEBM score
Number of dtudy participants
Number of knees
Mean age of patients
UKA design used
Number of UKAs in intervention group (young)
Number of UKAs in control group (old)
Age group classification
Follow-up duration
Number of revisions in intervention group (young)
Number of revisions in control group (old)
Revision rate in intervention group (young)
Revision rate in in control group (old)
KSS
OKS
Statistical tests used
Study conclusion
Other notes
